# Implications of a Self-Care Management Tool on Cardiovascular Events in Heart Failure Patients Within Six Months in a Regional City Hospital of Japan

**DOI:** 10.7759/cureus.89080

**Published:** 2025-07-30

**Authors:** Takumi Kawamura, Kazufumi Kitagaki, Miho Takeuchi, Kaori Arishima, Tsuyoshi Katou, Keisuke Watanabe

**Affiliations:** 1 Department of Rehabilitation, Shinbeppu Hospital, Federation of National Public Service Personnel Mutual Aid Associations, Beppu, JPN; 2 Faculty of Rehabilitation, Shijonawate Gakuen University, Daitō, JPN; 3 Department of Nursing, Shinbeppu Hospital, Federation of National Public Service Personnel Mutual Aid Associations, Beppu, JPN; 4 Department of Cardiology, Shinbeppu Hospital, Federation of National Public Service Personnel Mutual Aid Associations, Beppu, JPN

**Keywords:** cardiovascular events, heart failure, patient education, readmission, retrospective cohort study, self-care management tool

## Abstract

Background

Patients with heart failure (HF) often face the need to maintain stable symptoms after discharge. Self-care management, including symptom monitoring, is recommended; however, because of factors such as population aging, these self-care behaviors are difficult to maintain for some patients, and the re-hospitalization rate remains high in Japanese registries.

Objective

In this retrospective cohort study, we aimed to verify whether the use of a self-care management tool among hospitalized patients with HF in a regional city hospital contributes to the reduction of cardiovascular events within a period of six months.

Methods

We retrospectively analyzed 117 patients hospitalized and treated for HF in Japan between December 2020 and November 2022. Patients admitted by November 2021 were assigned to the non-intervention group, while those admitted after this date and eligible to use the tool were assigned to the intervention group. The incidence of readmission owing to cardiovascular events within six months after discharge was analyzed.

Results

The Gray test, with unexpected readmissions and deaths due to causes other than cardiovascular events as competing risks, indicated that the intervention of using the self-care management tool reduced cardiovascular events (p=0.045). Even after adjusting for the Meta-analysis Global Group in Chronic Heart Failure (MAGGIC) score, the logarithm of B-type natriuretic peptide, and the use of sodium-glucose cotransporter 2 inhibitors, the effect of intervention with the self-care management tool remained significant (hazard ratio: 0.33; 95% confidence interval: 0.15-0.74, p<0.01).

Conclusions

The use of a self-care management tool for patients with HF was associated with a reduction in cardiovascular events.

## Introduction

The number of patients with heart failure (HF) worldwide was 64.3 million by 2017, posing a significant public health concern [1]. Over recent decades, the prognosis of HF has slightly improved, but high hospitalization rates remain a significant issue [2]. Nearly a quarter of patients with HF are reported to have readmissions within 30 days after discharge, and approximately half are readmitted within six months, indicating that a significant number of patients experience early readmissions after discharge [3].

Patient factors are considered the major cause [4], and disease management, including symptom monitoring, is recommended in the 2025 guidelines of the Japanese Circulation Society and the Japanese Heart Failure Society Joint Working Group [5]. The American Heart Association recommends two self-care behaviors to prevent re-hospitalization in patients with HF: self-care maintenance and self-care management [6]. Self-care maintenance is defined as engaging in preventive behaviors, such as adherence to medication, reducing the amount of salt consumed in the diet, and actively monitoring symptoms and signs, while self-care management is defined as making decisions about symptoms and signs. However, because of factors such as population aging, these self-care behaviors are difficult for some patients to maintain [7], and indeed, the re-hospitalization rate remains high in Japanese registries [8-10]. In addition to individual patient factors, social background is an important but often overlooked determinant of risk stratification and clinical outcomes. Social determinants of health, including economic status, living conditions, social support, and access to healthcare resources, significantly influence patients’ ability to engage in effective self-care and affect their health outcomes. This perspective is increasingly recognized in the medical community [11].

To address this problem, Nakane et al. developed a self-care maintenance tool for patient education and a self-care management tool that enables self-care management by scoring weight and symptoms [7]. As a result of self-management with the tool, they reported a significantly lower cumulative one-year incidence of HF hospitalization. However, this study was conducted in an urban area, and there may be differences in age groups [12], frailty rates [13], and other social backgrounds [14-16] compared with the Oita Prefecture, where our hospital is located. These factors may affect heart failure self-management and potentially influence patient outcomes [5]. In fact, differences in heart failure readmission rates between urban and rural areas have been reported [17]. Therefore, the purpose of this retrospective cohort study was to evaluate whether the intervention using the self-care management tool during hospitalization in a regional city hospital reduces the incidence of cardiovascular events (readmission due to HF or arrhythmias, or sudden cardiac death or stroke) within six months in patients hospitalized for HF and to examine its potential effect on early readmission.

## Materials and methods

This was a single-center retrospective cohort study conducted at the Department of Cardiology, Shinbeppu Hospital, Beppu, Oita, Japan, between December 2020 and November 2022. The study was approved by the Ethics Committee of Shinbeppu Hospital (approval number: 2023003).

Eligibility criteria

The study included patients who were hospitalized and treated for HF between December 2020 to November 2022. Patients who died during hospitalization, could not be followed up for six months after discharge (thereafter visited other hospitals), were transferred to other hospitals, had missing data (i.e., cases with missing values related to confounding factors such as the Meta-analysis Global Group in Chronic Heart Failure (MAGGIC) score, B-type natriuretic peptide (BNP), and sodium-glucose cotransporter 2 inhibitors (SGLT2-i)) were excluded. The self-care management tool was implemented from December 2021 onwards, and patients who were hospitalised and treated for HF between December 2021 and November 2022 but did not have the self-help tool intervention were excluded from the study.

Study process

Patients were divided into two groups. Patients who were hospitalized from December 2020 to November 2021, when the self-care management tool intervention was not available, were classified as the non-intervention group. Patients who were hospitalized from December 2021 to November 2022 with the self-care management tool intervention were classified as the intervention group. All information was collected from the patient’s medical records, and all data were de-identified prior to analysis to ensure patient confidentiality.

Self-care management tool

The self-care management tool used in this study was the same as that developed and reported by Nakane et al. [7], who demonstrated its effectiveness in reducing HF hospitalizations in a cohort from an urban hospital setting. This tool quantifies weight and symptoms to guide decisions on when to seek medical attention. Points are assigned as follows: 1 point for any early symptoms of HF (e.g., exertional dyspnea, edema, cough, or loss of appetite), 3 points for a deviation from the preset weight, 4 points for a heart rate exceeding 120 beats per minute, and 5 points for the presence of dyspnea at rest. The total score is then calculated. Patients were instructed to visit an outpatient clinic within one week if the total score was 3 points, to seek medical attention on the same day or the following day if the score was 4 points, and to visit the emergency department if the score was 5 points or higher.

The self-care management tool was implemented as an intervention during hospitalization, and for patients with cognitive impairment, it was explained and introduced to their caregivers. The medical staff consulted with the attending physician about the intervention at a stage when HF symptoms had subsided (e.g., after oxygen withdrawal), and the physician made the decision to proceed with the intervention. Medical staff certified as a Certified HF Educator or nurse provided one-on-one guidance on the use of the system. From the time of instruction until discharge, medical staff assessed patients’ understanding and use of the tool on a daily basis. For patients with cognitive impairment, the tool was explained and introduced not only to the patients themselves but also to their caregivers. In this study, out of 40 patients in the intervention group, nine received the intervention through their caregivers.

Data collection

Outcomes

The primary outcome was defined as the occurrence of cardiovascular events (a composite event of readmission and death due to cardiovascular events) within six months after discharge. Cardiovascular events were defined as readmission due to HF or arrhythmias, sudden cardiac death, or stroke. The occurrence of events was set as the endpoint, and the number of days from discharge to the occurrence of the event was calculated. If six months had passed or if a scheduled hospitalization occurred, the observation was censored, and follow-up was completed.

Demographic Information

We obtained data on age, sex, and body mass index (BMI).

Clinical Patient Characteristics

We obtained data on the following comorbidities: hypertension, dyslipidemia, atrial flutter/atrial fibrillation, diabetes mellitus (DM), chronic kidney disease, chronic obstructive pulmonary disease (COPD), and anemia. Anemia was determined based on the World Health Organization (WHO) definition (hemoglobin (Hb) levels below 13 g/dL for male and below 12 g/dL for female patients). Additionally, New York Heart Association (NYHA) classification, the presence of a cardiovascular implantable electronic device, history of HF hospitalization, duration of HF, smoking history (current smoking status), living alone, certification in national long-term care insurance and its level, the presence of dementia, systolic blood pressure (SBP), walking independence, and the clinical frailty scale (CFS) were all collected. CFS was evaluated on a nine-point scale using the Japanese version of the Japanese Geriatrics Society CFS scale [18], and from previous studies; a level of 5 or higher was defined as frail, reflecting difficulty in performing instrumental activities of daily living. This threshold (CFS ≥5) has been commonly adopted in previous studies and is associated with poorer outcomes in older adults. Its validity has been demonstrated across various patient populations, including those admitted to intensive care units and hospitalized older individuals [19,20].

Blood Samples

We obtained data on plasma BNP, Hb, serum creatinine (Cr), estimated glomerular filtration rate (eGFR), and albumin. Following the formula from the Japanese Society of Nephrology [21], eGFR was calculated as follows: for males, eGFR = 194 × Cr^-1.094^ × age^-0.287^; for females, eGFR = 194 × Cr^-1.094^ × age^-0.287^ × 0.739.

Echocardiography

We estimated left ventricle ejection fraction (LVEF) using either the Teichholz or the modified Simpson method.

Medication

We obtained data regarding the presence of angiotensin-converting enzyme inhibitors (ACE-i), angiotensin receptor blockers (ARB), angiotensin receptor neprilysin inhibitors (ARNI), β-blockers, mineralocorticoid receptor antagonists, *SGLT2-i*, cardiac glycosides, diuretics (loop, thiazide, tolvaptan), 3-hydroxy-3-methylglutaryl coenzyme A reductase inhibitors (statins), and calcium channel blockers. In this study, ARNI was treated as the same classification as ACE-i/ARB.

Self-Management

We obtained data on the occurrence of unscheduled medical visits and telephone consultations for the self-management evaluation.

MAGGIC Score

The MAGGIC score was calculated based on 13 variables, including age, sex, SBP, BMI, duration of HF, current smoking, LVEF, NYHA classification, Cr, comorbidities such as COPD and DM, and the use of β-blockers and ACE-i/ARB [22].

Statistical analysis

Continuous variables were expressed as median (interquartile range* *(IQR)), and categorical variables were expressed as the number of patients (percentages). Comparisons of patient characteristics between the two groups were conducted using the Mann-Whitney U test for continuous variables and the Fisher's exact test for categorical variables as appropriate. For survival analysis, competing risks were unexpected rehospitalization and death from causes other than cardiovascular events, censored at the time of their occurrence, and the cumulative incidence of cardiovascular events between the two groups was investigated using the cumulative incidence function and Gray’s test. Additionally, we also observed an association between self-care management tools and cardiovascular events after adjusting for potential confounders of cardiovascular events in the Fine-Gray model, with the Schoenfeld residuals method, indicating that the proportional subdistribution hazards assumption was met. Confounders were adjusted for MAGGIC score, logarithm of BNP (log BNP), and use of SGLT2-i. We adopted a complete-case analysis approach, excluding patients with missing data for MAGGIC score, BNP, or SGLT2-i from the analysis. The MAGGIC score is a risk score that predicts one-year and three-year all-cause mortality in patients with HF and has shown moderate discrimination and good calibration in Japan. Furthermore, adding log BNP values to the MAGGIC score variables has been shown to improve discriminatory power[23]. Statistical analyses were performed using EZR version 1.63 [24], and a p-value <0.05 was considered statistically significant.

## Results

Patient characteristics

**Figure 1:**
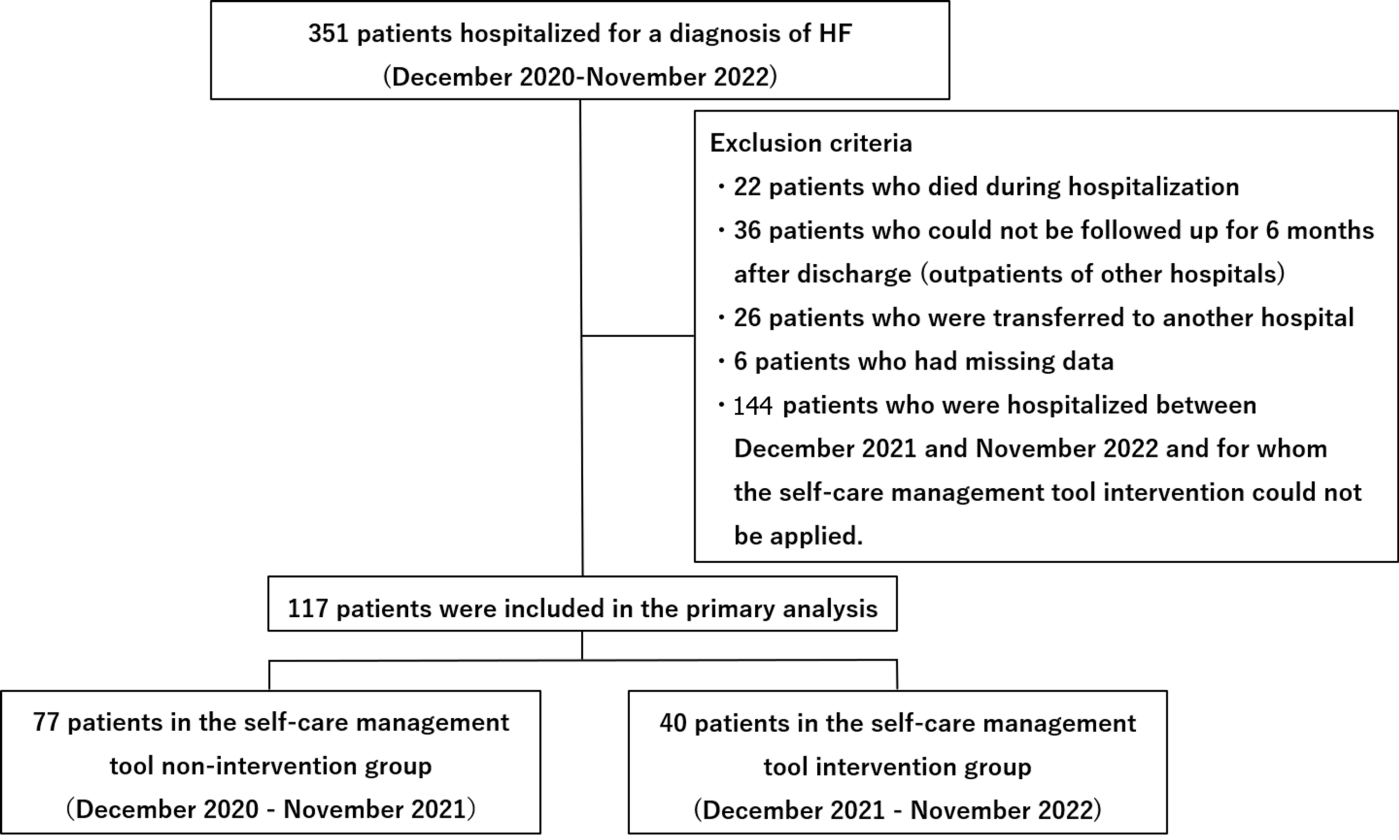
Flowchart of patient selection

A patient selection flowchart is presented in Figure 1. Of the 117 patients who met the eligibility criteria, 77 were included in the non-intervention group and 40 were included in the intervention group (Figure 1).

Table 1 shows the patient characteristics. The median age of the patients was 83 years (IQR, 76～88), 56% were male, and the median BMI was 21.1 kg/m² (IQR, 19.2～24.1). The median LVEF was 45% (IQR, 30～65), with 43% of patients classified as having HFrEF. Frailty was observed in 58 patients (49.6%). The proportion of patients with HFrEF and the use of ARNI and SGLT2-i were higher in the intervention group, while other characteristics were not significantly different between groups. The tool was introduced at a median of 15 days (IQR, 8～18) after admission.

**Table 1:** Baseline patient characteristics ^a ^Mann–Whitney U test, ^b ^Fisher's exact test BMI: Body Mass Index, NYHA: New York Heart Association class, HT: Hypertension, DLP: Dyslipidemia, AFL/AF: Atrial flutter/Atrial fibrillation, DM: Diabetes Mellitus, CKD: Chronic Kidney Disease, COPD: Chronic Obstructive Pulmonary Disease, BNP: B-type Natriuretic Peptide, Hb: Hemoglobin, eGFR: estimated Glomerular Filtration Rate, Alb: Albumin, LVEF: Left Ventricle Ejection Fraction, HFrEF: Heart Failure with reduced Ejection Fraction, ACE-ii Angiotensin-Converting Enzyme inhibitors, ARB: Angiotensin Receptor Blockers, ARNI: Angiotensin Receptor Neprilysin Inhibitors, RAAS: Renin-Angiotensin-Aldosterone System inhibitors, MRA: Mineralocorticoid Receptor Antagonists, SGLT-2i: Sodium-Glucose cotransporter 2 inhibitors, CCB: Calcium Channel Blockers, CIED: Cardiac Implantable Electronic Device, CFS: Clinical Frailty Scale, MAGGIC score: Meta-Analysis Global Group in Chronic Heart Failure score

	All (N=117)	Intervention group (n=40)	Non-intervention group (n=77)	Test statistics	ｐ-value
Age (years), median (IQR)	83 (76～88)	81.5 (75～87)	84 (78～88)	U =1675.5	0.44^a^
Sex (male), n (%)	66 (56.4)	21 (52.5)	45 (58.4)	N/A	0.56^b^
BMI (kg/㎡), median (IQR)	21.1 (19～22.8)	21.4 (19.0～24.4)	21.0 (19.3～22.3)	U =1328	0.22^a^
NYHA ≧Ⅱ, n (%)	101 (86.3)	32 (80.0)	69 (89.6)	N/A	0.17^b^
Comorbidity, n (%)					
HT	95（81.2）	36（90.0）	59（76.6）	N/A	0.09^b^
DLP	50（42.7）	22（55.0）	28（36.4）	N/A	0.08^b^
AFL/AF	62（53.0）	20（50.0）	42（54.5）	N/A	0.7^b^
DM	52（44.4）	18（45.0）	34（44.2）	N/A	1^b^
CKD	37（31.6）	12（30.0）	25（32.5）	N/A	0.84^b^
COPD	2（1.7）	1（2.5）	1（1.3）	N/A	1^b^
Anemia	80（68.4）	29（72.5）	51（66.2）	N/A	0.54^b^
Laboratory data, median (IQR)					
BNP (pg/mL)	277.6（141～570）	331.0（178～505）	269.0（118～604）	U=1418.5	0.49^a^
Hb (g/dL)	11.5（10.2～13.1）	11.2（10.2～12.7）	11.6（10.2～13.1）	U=1578.5	0.83^a^
Cr (mg/dL)	1.24（0.99～1.67）	1.27（1.00～1.55）	1.22（0.98～1.76）	U=1519.5	0.91^a^
eGFR (ml/min/1.73㎡)	38.5（26.2～51.2）	40.5（26.2～49.0）	37.8（26.2～52.3）	U=1587.5	0.79^a^
Alb (g/dL)	3.5（3.3～3.8）	3.6（3.4～3.8）	3.5（3.2～3.8）	U=1390	0.59^a^
LVEF (%), median (IQR)	45（30～65）	38（30～61）	50（35～65）	U=1831.5	0.09^a^
HFrEF, n (%)	43（36.8）	20（50.0）	23（29.9）	N/A	0.04^b^
Medication, n (%)					
ACE-i	18（15.4）	3（7.5）	15（19.5）	N/A	0.11^b^
ARB	23（19.7）	5（12.5）	18（23.4）	N/A	0.22^b^
ARNI	21（17.9）	15（37.5）	6（7.8）	N/A	<0.01^b^
RAAS	62（53.0）	23（57.5）	39（50.6）	N/A	0.56^b^
β-blocker	77（65.8）	25（62.5）	52（67.5）	N/A	0.68^b^
MRA	52（44.4）	17（42.5）	35（45.5）	N/A	0.85^b^
SGLT2-i	28（23.9）	18（45.0）	10（13.0）	N/A	<0.01^b^
Inotropic agents	19（16.2）	8（20.0）	11（14.3）	N/A	0.44^b^
Loop diuretics	97（82.9）	32（80.0）	65（84.4）	N/A	0.61^b^
Thiazide	18（15.4）	6（15.0）	12（15.6）	N/A	1^b^
Tolvaptan	40（34.2）	14（35.0）	26（33.8）	N/A	1^b^
Statin	55（47.0）	24（60.0）	31（40.3）	N/A	0.05^b^
CCB	37（31.6）	11（27.5）	26（33.8）	N/A	0.54^b^
CIED	14（12.0）	4（10.0）	10（13.0）	N/A	0.77^b^
Past HF hospitalization	58（49.6）	23（57.5）	35（45.5）	N/A	0.25^b^
Smoking history	63（53.8）	22（55.0）	41（53.2）	N/A	1^b^
Living alone	27（23.1）	13（32.5）	14（18.2）	N/A	0.11^b^
Certification in national long- term care insurance	38（32.5）	14（35.0）	24（31.2）	N/A	0.68^b^
≧Care level	29（24.8）	12（30.0）	17（22.1）	N/A	0.37^b^
Dementia	18（15.4）	4（10.0）	14（18.2）	N/A	0.29^b^
Walking independence	93（79.5）	32（80.0）	61（79.2）	N/A	1^b^
Frailty	58（49.6）	18（45.0）	40（51.9）	N/A	0.56^b^
Unplanned ambulatory visits and telephone consultation	20（17.1）	9（22.5）	11（14.3）	N/A	0.31^b^
MAGGIC score, median (IQR)	29（25～33）	29（25～34）	28（26～32）	U=1512.5	0.88^a^

Cumulative incidence

**Figure 2:**
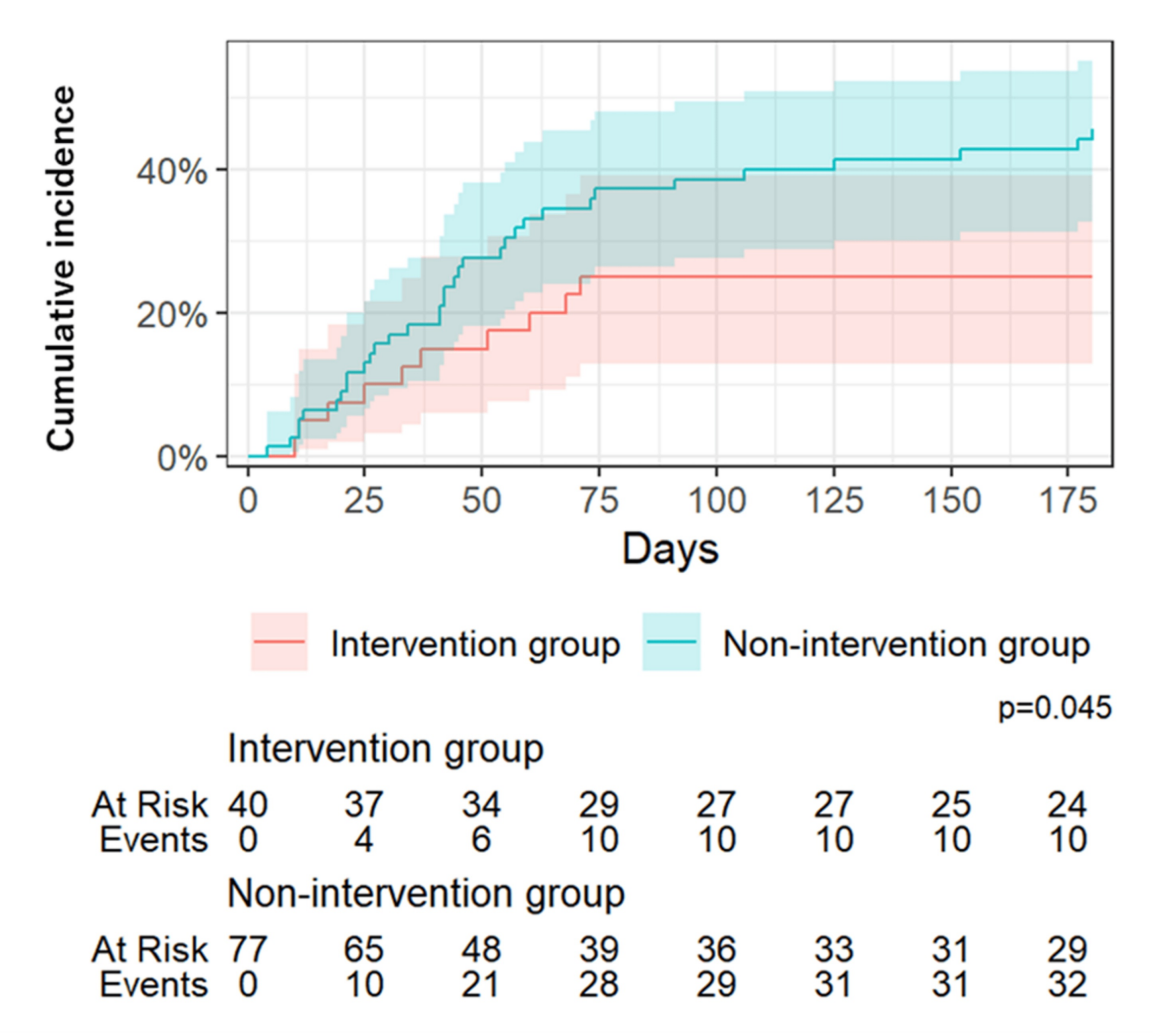
Cumulative incidence of cardiovascular events with and without the self-care management tool

The mean observation period was 111 ± 69 days, and cardiovascular events were observed in 10 patients in the intervention group and 34 patients in the non-intervention group. Cardiovascular events included 10 cases of HF in the intervention group, 33 cases of HF in the non-intervention group, and one case of loss of consciousness due to atrial fibrillation. Sudden cardiac death or stroke was not observed. The median time to the occurrence of cardiovascular events was 180 days in the intervention group and 77 days in the non-intervention group. The cumulative incidence of cardiovascular events was significantly lower in the intervention group than in the non-intervention group (χ2=4.1, p=0.043) (Figure 2). The absolute risk reduction was 19.2%, and the number needed to treat was approximately 5, indicating that the self-care management tool prevented one cardiovascular event for every five patients treated.

Fine-Gray model

Assessment of proportional subdistribution hazards using Schoenfeld‑type residuals showed no evidence of time‑dependent effects for any covariate (all p > 0.05), confirming that the proportionality assumption was satisfied. Adjusted for MAGGIC score, log BNP, and the use of sSGLT2-i, the hazard ratio (HR) for the occurrence of cardiovascular events with the intervention of the self-care management tool was 0.33 (95% confidence interval (CI) 0.15-0.74, p<0.01) (Table 2).

**Table 2:** Hazard ratios and 95% confidence intervals for cardiovascular event events using the Fine-Gray model CI: Confidence Interval, HR: Hazard Ratio, log BNP: logarithm B-type Natriuretic Peptide, MAGGIC score: Meta-analysis Global Group in Chronic Heart Failure Score, SGLT2-i: Sodium-Glucose Cotransporter 2 Inhibitor

Group	HR	95% CI	Test statistics	p-value
Non-intervention group	—	—		
Intervention group	0.33	0.15-0.74	Z =－2.70	<0.01
MAGGIC score	1.10	1.05-1.16	Z=3.67	<0.01
log BNP	2.06	0.99-4.27	Z=1.93	0.053
SGLT2-i	2.45	1.26-4.73	Z=2.66	<0.01

## Discussion

In this study, we investigated whether the intervention (using a self-care management tool) influences the reduction of cardiovascular events within six months after discharge in patients admitted to a regional city hospital with a diagnosis of HF, using a cumulative incidence function and the Fine-Gray model. The results showed that even in patients admitted to a regional city hospital, the rate of cardiovascular events within six months was significantly lower in the intervention group than in the non-intervention group, and this effect remained unchanged after adjustment for confounding factors. The results suggest that the self-care management tool may be effective in reducing cardiovascular events in a regional city hospital.

In this study, cardiovascular events within six months were observed in 39% of patients, a higher proportion than that reported by Nakane et al. [7]. There are various reports on the prognostic factors for patients with HF [10,25-27]. The patients in this study were older, with a median age of 83 years, and many had comorbidities. The number of patients with a history of hospitalization for HF was 23% in Nakane et al.'s study [7] and 50% in this study, suggesting a high frequency of HF hospitalization in this study cohort. In addition, the rate of frailty was also high, consistent with previous studies [28], despite differences in assessment methods, and these factors may have contributed to the high rate of cardiovascular events. The effect of the self-care management tool in reducing cardiovascular events in patients with HF in a regional hospital was consistent with the results of the previous study conducted in an urban area [7]. The results suggest that the use of self-care management tools in self-management may be effective in preventing cardiovascular events in patients with HF, also in a regional hospital setting.It has also been reported that the highest rate of rehospitalization due to HF occurs within three months after discharge from the hospital [29]. The results of this study also showed that 86% of the cardiovascular events occurred within three months. The intervention using the self-care management tool can be expected to reduce the incidence of cardiovascular events in the early stages of HF.

Various factors that prevent cardiovascular events are associated with self-care management. It is estimated that more than 50% of HF exacerbations are caused by patient factors, such as salt and fluid restriction and inadequate use of therapeutic drugs [4]. Appropriate self-care in patients with HF plays an important role in preventing exacerbations of HF, and meta-analyses have shown that self-care improves prognosis [30]. In Japan, self-care has been reported to reduce cardiac events, and patient education focusing on self-care behaviors is essential because HF knowledge is associated with self-care compliance [31]. The self-care management tool enables both self-care maintenance and self-care management, and it is possible that self-care maintenance, which includes medication adherence and preventive behaviors such as salt restriction, improves first through patient education. While self-care management is often evaluated through responses to emerging symptoms [6], such as early consultation behavior [7], this study did not find a statistically significant difference in the rate of unscheduled outpatient visits or telephone consultations between the two groups. However, the intervention group showed a higher proportion (22.5%) than the non-intervention group (14.3%), which may suggest increased patient engagement. Nevertheless, since the difference was not statistically significant and no process indicators, such as adherence scores or tool usage frequency, were collected, the observed behavior should be interpreted with caution. Further studies are needed to clarify whether the observed trends in consultation behavior reflect meaningful improvements in self-care or are associated with improved clinical outcomes.

Limitations and future prospects

The limitations of this study include the following: First, this was a single-center, retrospective cohort study with a small sample size, and selection and information bias may have been introduced because of the exclusion of patients who were not provided with the self-care management tool between December 2021 and November 2022. Also, since group allocation in this study was based on the date of hospitalization, the possibility that biases related to changes in healthcare delivery or other unmeasured confounders influenced the results cannot be ruled out. Additionally, as this was a retrospective observational study, outcome assessors were not blinded to group assignment. Since certified HF educators who delivered the intervention were also involved in outcome evaluation, the possibility of performance and detection bias cannot be excluded. Second, there may be confounding biases stemming from differences in patient characteristics and insufficient adjustment for important prognostic factors owing to the risk of overadjustment or the influence of unmeasured confounders. Therefore, caution is warranted in interpreting and generalizing the findings. Third, the quality of self-care itself was not assessed, and the mechanism by which the use of the self-care management tool reduces cardiovascular events remains unclear. In this study, the usage of the self-care management tool during hospitalization was assessed; however, data on patient adherence or active engagement with the tool after discharge were not available and were therefore not included in the analysis. Moreover, important factors such as cognitive impairment and whether the intervention was delivered to the patient or their caregiver were not evaluated. Further studies are required to investigate these factors and to clarify what contributes to the sustained effectiveness of the self-care management tool. Finally, since this study was conducted in a single regional hospital in Japan, the findings may have limited generalizability to other settings or populations.

## Conclusions

The results of this retrospective cohort study suggest that the use of a self-care management tool may be associated with a reduction in cardiovascular events within six months after discharge among patients with HF at a regional hospital. This association remained significant after adjustment for confounding factors. Despite these limitations, the findings imply potential clinical relevance of implementing self-care management tools in practical, resource-limited regional healthcare settings. Further prospective and larger-scale studies are warranted to validate these findings and to elucidate the mechanisms by which self-care interventions may improve clinical outcomes.
